# Extraction of use case diagram elements using natural language processing and network science

**DOI:** 10.1371/journal.pone.0287502

**Published:** 2023-06-23

**Authors:** Maryam Imtiaz Malik, Muddassar Azam Sindhu, Rabeeh Ayaz Abbasi

**Affiliations:** 1 Department of Computer Science, Quaid-i-Azam University, Islamabad, Pakistan; 2 Department of Software Engineering, National University of Modern Languages, Islamabad, Pakistan; The University of Lahore, PAKISTAN

## Abstract

Software engineering artifact extraction from natural language requirements without human intervention is a challenging task. Out of these artifacts, the use case plays a prominent role in software design and development. In the literature, most of the approaches are either semi-automated or necessitate formalism or make use of restricted natural language for the extraction of use cases from textual requirements. In this paper, we resolve the challenge of automated artifact extraction from natural language requirements. We propose an automated approach to generate use cases, actors, and their relationships from natural language requirements. Our proposed approach involves no human intervention or formalism. To automate the proposed approach, we have used Natural Language Processing and Network Science. Our proposed approach provides promising results for the extraction of use case elements from natural language requirements. We validate the proposed approach using several literature-based case studies. The proposed approach significantly improves the results in comparison to an existing approach. On average, the proposed approach achieves around 71.5% accuracy (F-Measure), whereas the baseline method achieves around 16% accuracy (F-Measure) on average. The evaluation of the proposed approach on the literature-based case studies shows its significance for the extraction of use case elements from natural language requirements. The approach reduces human effort in software design and development.

## Introduction

The software requirement specification is one of the initial tasks for the development of software. It provides the functionality of the system. These requirements are specified either informally, semi-formally, or formally. Informal requirements are expressed using natural language and are easily understandable by novice users. These requirements can be categorized as free text or restricted natural language requirements. The free text requirements are usually documented in an unstructured manner. They do not contain the use cases as in the Software Requirements Specification (SRS) document. Use cases are the primary source of communication among all stakeholders and provide the functionality of the system explicitly [1]. The applicability of use cases is found across several areas including big data [2, 3], software effort estimation [4], formal specification [5], augmented reality [6], e-commerce [7], health care [8], safety critical systems and blockchain [9]. The wider range of its applicability is the source of motivation for us. The generation of use cases from the free text requirements requires human effort, time and cost.

The literature reveals several approaches to generate UML diagrams and use cases [10–17]. However, most of the approaches require restricted natural language, formalism, or are semi-automated. Therefore, it is desirable to generate use cases and identify actors and relationships automatically, without depending on formalism, restricted natural language, or additional details. In this paper, we provide a set of algorithms to generate use cases, actors and their relationships automatically from natural language requirements using Natural Language Processing (NLP) and Network Science.

NLP and Network Science have a broad range of applicability including text classification [18], Gatekeeping on Twitter [19], toxic comment classification [20], Rebels Identification [21], analysis of public discussions [22], social network infodemic [23], detection of mental illness [24], and role of ChatGPT in health care [25].

The main contributions of our paper are as follows:

- We have devised a set of algorithms to extract actors, use cases and relationships from textual requirements. Our approach extracts both primary and external actors.
- Our proposed approach uses NLP and Network Science for the extraction of elements and visualization of the graph.
- Our proposed approach is validated on the literature-based case studies showing its significance in comparison with the state-of-the-art approach.
- We have also provided the code along with a demonstration video to reproduce the results.

We have used Precision, Recall and F-Measure for the evaluation of results on literature-based case studies. Our approach yields significant results in comparison with the state-of-the-art approach using NLP and Network Science techniques. We identify the actors from the textual requirement in multiple phases to strengthen the approach. Our proposed approach uses both noun and verb for the identification of use cases.

The rest of the paper is organized as follows: Related work section reviews the literature which is followed by the proposed approach. In the Experimental Setup section, we elaborate the experimental setup along with the details of case studies. The result analysis and discussion section presents the results and compares them with an existing approach. We also provide the threats to validity section. Lastly, we conclude the paper.

## Related work

This section provides an extensive literature review on the use case extraction from natural language requirements.

We discuss the literature review in three broad categories. These categories include machine learning and artificial neural network (ANN), NLP, the recursive object model (ROM) and rule-based approaches. However, most of the approaches in each category also require a set of rules to generate use cases and actors.

### Machine learning and artificial neural network

Imam et al. [26] propose an approach to extract software entities from natural language requirements. These include: use cases, actors and the system. The software entities are extracted using the SVM machine learning approach. The tokens of each sentence are annotated with the linguistic attributes both at the training and testing phase.

Kochbati et al. [10] propose an approach to generate use cases from the user stories. The approach involves word and requirement level semantic similarity between the requirements. It also clusters similar requirements together. The use case models are generated from the labeled clusters using defined rules.

Tiwari et al. [27] propose an approach to generate use cases and actors using machine learning and NLP techniques.

Osman et al. [28] propose an approach for the generation of use cases from natural language requirements using machine learning. The authors’ main focus is to increase the accuracy and speed of the approach. The requirements are categorized into new and old data. Old data is used as testing data and new data is pre-processed for the generation of the use case diagram. The results generated from the use case diagram are fed into the machine learning algorithm(s) for prediction. If the predicted results and the results extracted from the generated use case diagram are the same then it shows 100% accuracy. However, the authors do not discuss the technique for the generation of use cases.

Al-Hroob et al. [11] propose a semi-automated approach for the generation of actors and actions i.e use cases from natural language requirements. The approach syntactically and semantically analyzes each word and then assigns a numeric code to each word, POS tag and thematic role. The numeric data is fed into the back propagation neural network to generate actors and actions. The approach evaluates using five case studies.

Moketar and Kamalrudin [29] propose an approach for the generation of essential use case models (EUC) from textual requirements. The EUC model is generated using rule-based, clustering, and classification techniques. They define three sentence structures for the extraction of phrases. The extracted EUC is matched with the synonyms using WordNet and domain dictionaries. These EUC phrases are stored in the pattern library for the extraction of the EUC model.

Narawita et al. [12], Narawita and Vidanage [13] propose an approach to generate use case and class diagram from natural language requirements. The approach identifies the actors, classes and use cases using syntactical analysis and word chunking techniques. The Weka module is used to classify the use cases and class relationships. The evaluator of an approach specifies the limitation that the generation of actors using nouns through the rule-based approach may not provide accurate results for all scenarios. It removes unwanted actors manually. It is also used to rate the extracted use cases.

Vemuri et al. [30] propose an approach to generate the use case diagram from the textual requirements document. This approach uses a supervised learning method for the classification of actors and use cases. The nouns in the subject part are used to train for the actors. The verbs and nouns in the predicate-object part of the sentence are used to train for classification of use cases. Naive Bayes classifier is then used for the classification of actors and use cases. The approach also has pre and post-processing techniques.

### Natural language processing

M. Maatuk and A. Abdelnabi [31] propose an approach to generate the use case and activity diagrams from natural language requirements. The NLP techniques and defined rules generate use cases and activity diagrams. These include: tokenization, stemming, lemmatization, typed dependency, and recognition of grammatical relations as NLP techniques. The textual requirements need to be normalized or written using sixteen defined rules for the generation of UML diagrams. M. Maatuk and A. Abdelnabi [31] encourage us to use OpenIE CoreNLP for the triplet generation and exclude the word “system” from an actor. However, our approach has used OpenIE 5.0 and OpenIE CoreNLP to generate accurate triplets. Our approach has also used network science, semantic similarity along with NLP techniques. We use both the predicates and objects to generate use cases. Further, our approach does not require type dependency to identify subject tags. We have only used the OpenIE for the generation of triplets.

Hamza and Hammad [32] propose an approach to generate use cases from natural language requirements. This approach uses Part of Speech (POS) tags, stemming, tokenization, grammatical knowledge patterns along with a set of rules for the generation process. The grammatical patterns for active and passive voice are used. The “subject” and “pronoun other than those that are written after a verb” are used to represent an actor. Further, the original system name is eliminated to represent external actors. The use cases are represented by verb and noun excluding the helping verbs. The relationship is identified through the relation of an actor with the use case in a particular sentence. This paper encourages to identify passive voice sentences using the word “by” and excludes the original system name from an actor.

Tiwari et al. [33] propose an approach to generate use case scenarios from natural language requirements’ documents. The approach uses semantic role labeling, POS tags and dependency tree for its generation process. The approach relies on the set of rules for the identification of use case scenarios. It identifies some irrelevant actors without the incorporation of question-based analysis. The authors do not discuss external actors. Further, the approach is dependent on “include” and “extend” keywords for the identification of include and extend use cases. The approach is evaluated using ten case studies quantitatively and qualitatively.

Jebril et al. [14] propose a semi-automated approach to identify actors and use cases from requirements using semantic role labeling techniques.

Elallaoui et al. [15] propose an approach for the generation of use cases, actors and relationships. These are generated only from user stories written in the Wautelet template. The approach uses some predefined nouns, verbs POS tags for the identification of actors and use cases. They evaluate their approach using one case study.

Gilson and Irwin [34] propose an approach to generate the robustness diagram from user stories. The approach has used named entity recognition, coreference resolution, and a dependency tree for the generation of the robustness diagram. The spacy library is used for performing NLP tasks.

Alksasbeh et al. [35] propose an approach for the generation of use cases from natural language requirements using syntactic and semantic analysis. Actors, use cases and relationships are identified using a set of rules.

Deeptimahanti and Sanyal [36] propose an approach to generate UML diagrams from the natural language requirements. The requirements are expressed in active voice and subject-predicate-object form. The requirements are preprocessed for the UML diagram generation. The actors are identified from the subject and object using noun phrases. Use cases are extracted using verb phrases. The preposition is used to provide the relationship between actors and use cases.

Sibarani et al. [37] propose a tool for the generation of actors, use cases and their responsibilities from natural language requirements written in subject-predicate-object (SPO) form. The tool has used the syntactic parser and pronoun resolver. An actor represents the first word of a sentence which is a noun. The verb and the two words after an actor are used to represent a use case. The sentences which correspond to some use cases are used as a responsibility of that particular use case.

Deeptimahanti and Babar [38] generate a use case and a class diagram. The sentences are represented in subject-object or subject-predicate-object form. The approach uses a set of rules along with NLP techniques for the generation process.

Bajwa and Hyder [39] propose an approach to generate the use case diagram using POS tagging, tokenization, and subject-action-object part of the sentence.

Kumar and Sanyal [40] propose an approach to generate use cases using named entity recognition, morphological analysis, pronoun resolution, and parsing. This approach splits the complex sentence into a single sentence, so that each sentence has a subject and a predicate. These sentences should be in active voice. Afterward, each sentence is parsed using the Stanford parser. The approach uses named entity recognition to join two adjacent nouns together. It represents actor and use cases using the noun phrases and verbs respectively. The relationship is identified using the preposition between noun and verb. The approach also generates class diagrams.

Cayaba et al. [41] discuss system architecture to generate use cases, actors, and relationships. It contains a parser, lexicon, discourse, and semantic analyzer. The lexicon contains business-specific terms.

Subramaniam et al. [16] uses a set of rules to extract use cases and actors. They have also used a glossary, dictionary, and Stanford parser for the generation process. Domain-specific words and English words with POS tags are stored in the glossary and dictionary respectively.

Vasques et al. [42] propose an approach to provide the relevant knowledge for use case extraction. This approach deals with different problems including redundancy, inconsistency, and incompleteness. The approach has used the Verbka technique. In this approach, the analyst prepared the data. The text preparation includes passive to active voice conversion, splitting the complex sentence into smaller ones, subject-action-object formation, etc. Afterward, the verbs are selected for the classification of syntagmas and assignment of thematic proto roles. Similar concepts are then clustered. The concept map is generated to identify cause-effect relationships. The concept map is in the form of a network with different colored edges. Each color represents a particular relationship. In this paper, the possible use cases are also provided with the semantically structured statements associated with a particular actor. These actors, actions, and use cases are used to provide a UML diagram. Vasques et al. [43] also conducted a study with students using this technique to check its performance.

### Recursive object model

Seresht and Ormandjieva [17] propose an approach to generate use cases from the requirements. The approach requires manual generation of the Expert Comparable Contextual (ECC) model to provide actors. The use cases are generated from the textual requirements using the Recursive Object Model (ROM). The ROM [44] is based on axiomatic theory. It represents the nodes and edges with various symbols depending upon the linguistic style. Although they use the primary actor and its corresponding edges for the generation, the process necessitates some restrictions in the textual requirement. The tool is validated with the models generated by experts on a case study.

Similarly, Wan et al. [45] have used ROM for the generation of SysML models. This model contains the use case, activity diagram, and block diagram. It generates triplets from the ROM diagram. It represents the actor either as a human noun in the subject, or there is a predicate relation of the verb with the main product along with the null subject. The use cases are represented by the verb object. Wan et al. [45] encourage us to use network science for the generation of use cases and actors. However, our approach generates the triplets directly from textual requirements and a network is formed using these triplets. No intermediate ROM diagram is required.

Further, the ROM is also used to generate use cases in the [46]. In their approach ROM is represented in XRD format. They use the graph traversal algorithm to extract the object. The highest priority noun object represents an actor. Action is extracted using the adjacency list of an actor. Chen and Zeng [46] also encourage the use of network science for the generation of use cases and actors.

### Rule-based approach

Nguyen et al. [47] propose an approach for the generation of goal use case model from the SRS document. The approach has used a set of rules for the identification of the goal use case model. Further, the textual requirements are polished and Mallet was used for goal classification. Similarly, a set of rules are used to extract use cases and actors from Arabic requirements in [48–50].

We have observed from the literature on use case identification that most of the approaches require restricted natural language, a set of keywords, and formalism for the identification of use cases, actors, and their relationship. Further, some of the approaches generate use cases and actors either manually or semi-automatically. However, some authors do not discuss the details for the generation of use cases, actors, or both.

## Proposed approach

**Fig 1:**
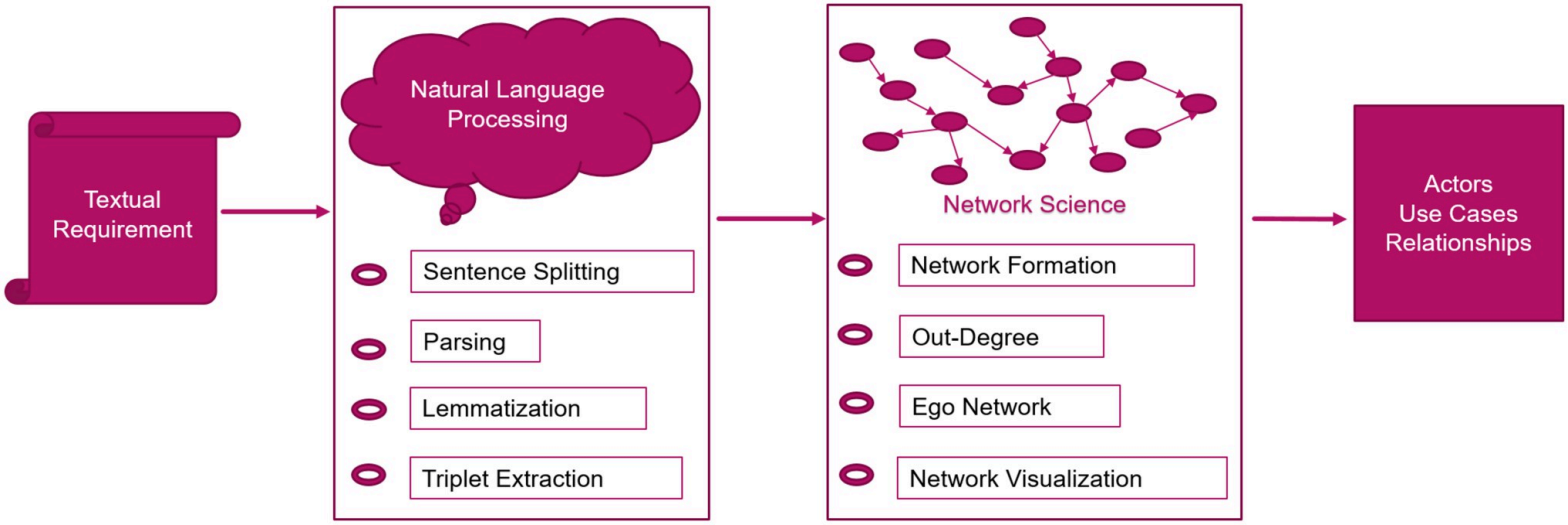
Proposed approach.

Our proposed approach generates use cases, actors, and relationships from natural language requirements. We use network science and natural language processing techniques (NLP). The NLP techniques include sentence splitting, parsing, lemmatization and triplet extraction. Our proposed approach identifies the actors from the textual requirement in multiple phases. Each phase improves the identification of actors. Fig 1, shows the higher level diagram of our proposed approach.

**Fig 2:**
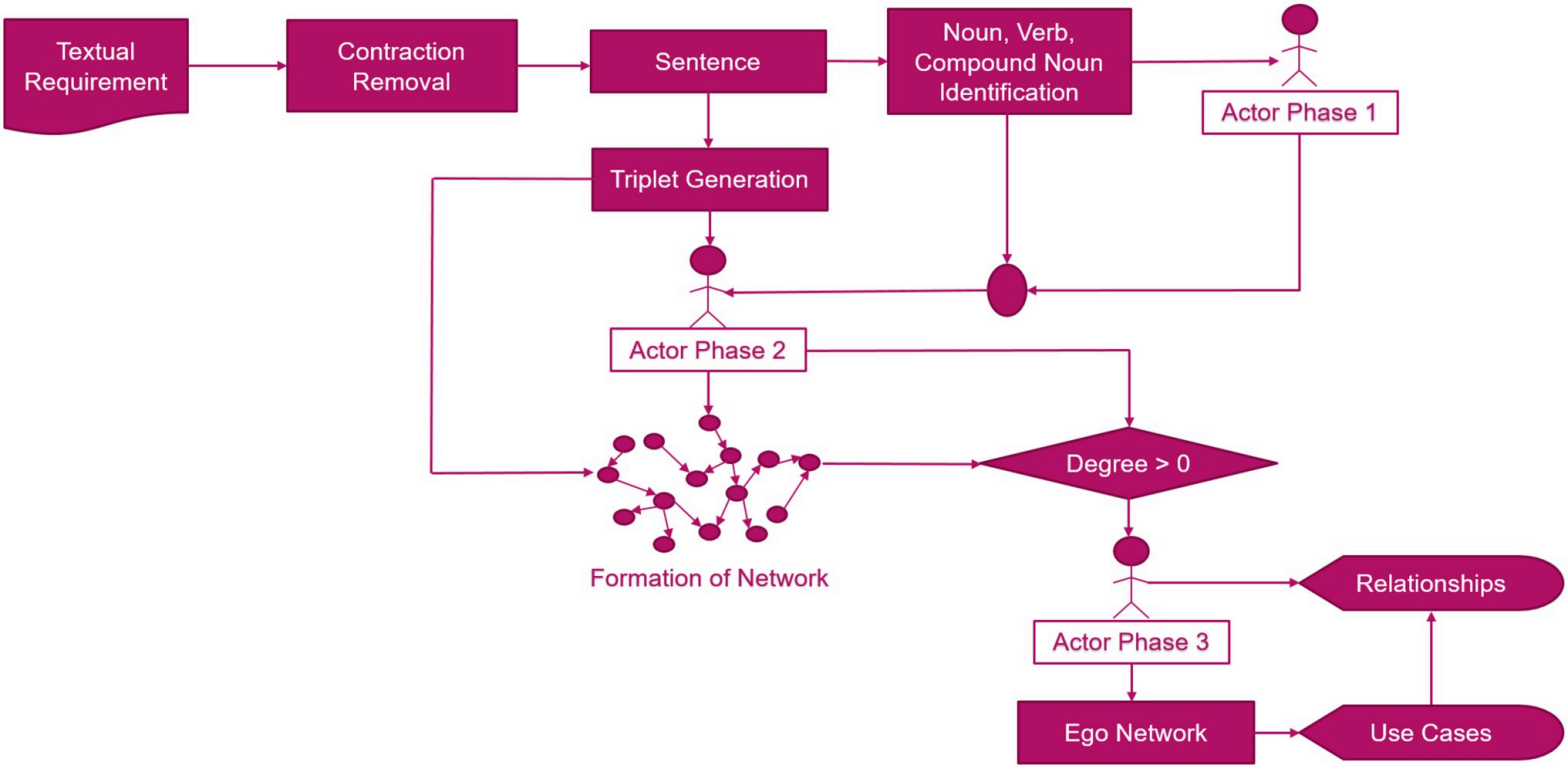
Process flow of proposed approach.

Our proposed approach takes textual requirements as input to identify use cases, actors and their relationships. Each sentence of textual requirement is split into a single sentence in Algorithm 1. We have identified the noun, verbs and compound nouns from each sentence in Algorithm 2. These nouns, verbs and compound nouns facilitate in the identification of actors and use cases in the later algorithms. The noun list of each sentence is used as an input in Algorithm 3 to identify the actors in phase 1. We have also identified the triplets from each sentence. These triplets are used as input in Algorithm 4 to identify the actors in phase 2. This algorithm is also used to identify the nodes and links of the graph. These actors and links are provided in Algorithm 1 for the formation of the network and the identification of use cases and the actors in the final phase.Fig 2, shows the process flow of our proposed approach.

**Fig 3:**
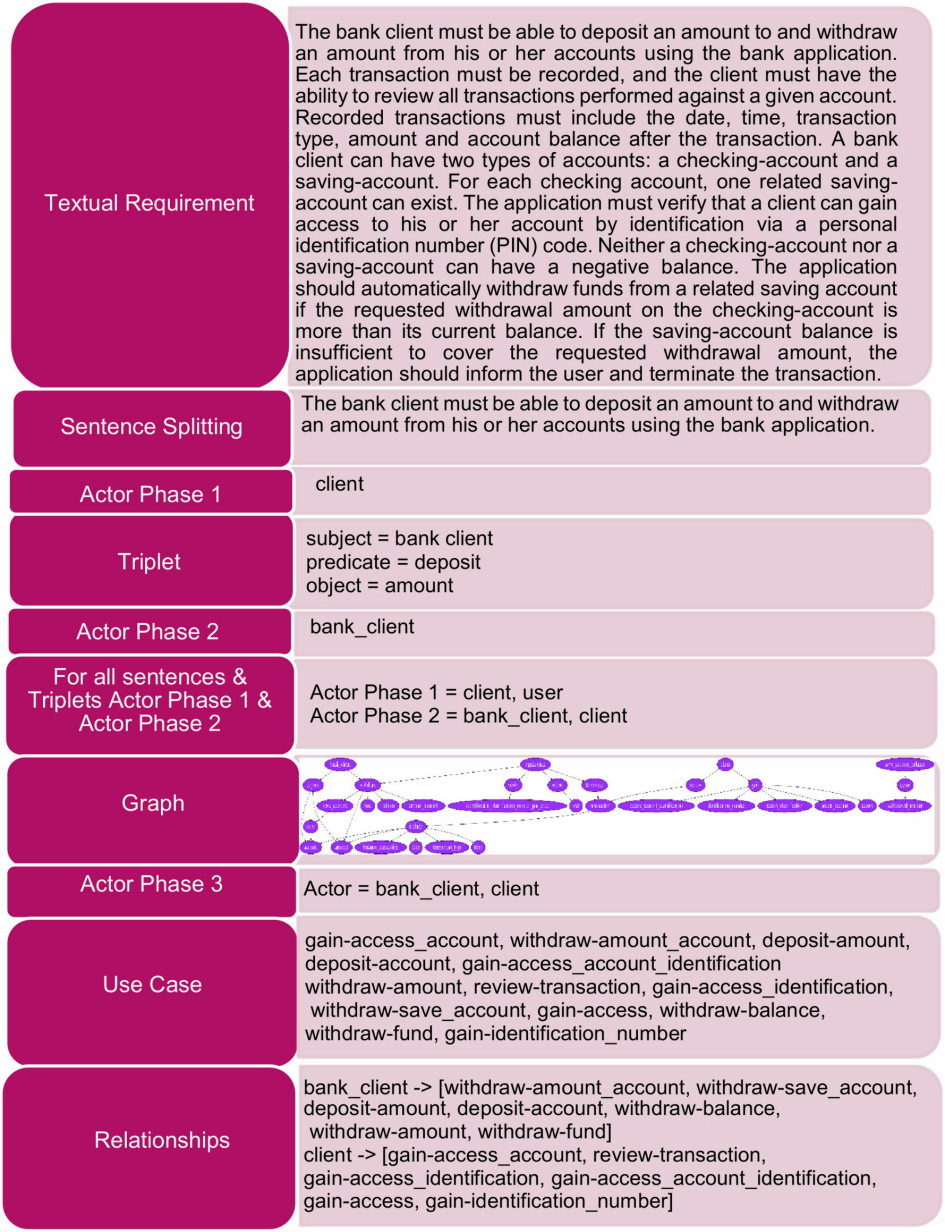
Example.

We have also illustrated some steps of our proposed approach with an example in Fig 3. The subsequent sections discuss the details of the proposed approach through Algorithms 1 to 5.

### Textual requirement

Our proposed approach generates the UML model from the textual requirements. These requirements can be written both in the active voice and passive voice. It can generate both primary and external actors.

### Preprocessing

The proposed approach detects and removes contractions from the textual requirements. We use a set of pairs for the contraction removal from https://github.com/dipanjanS/practical-machine-learning-with-python/blob/master/bonus%20content/nlp%20proven%20approach/contractions.py. There are two possibilities for the removal of contraction *‘d* as had or would. We distinguish these two words for the set of pairs using two conditions. These conditions are applied on the POS tag of *‘d* at the sentence level. If the POS tag is “modal” (MD) then the contraction is replaced by “would” whereas if the POS tag is a “verb in past tense” (VBD), it is replaced by “had”.

### Sentence splitting and triplet generation

We split each sentence of textual requirement as discussed in Algorithm 1. We identify nouns and verbs from each sentence using Algorithm 1. In our approach, the triplets are extracted from each sentence of textual requirement using Open Information Extraction (OpenIE). The OpenIE is used to extract subject, predicate and object in the form of triplets from the text. We observe that the single OpenIE system cannot generate accurate triplets for all kinds of sentences. Therefore, we use Open IE 5.0 and OpenIE-CoreNLP to extract more accurate triplets from each sentence of textual requirement. The Open IE 5.0 generates an output file containing the sentence, triple along with the confidence. We have preprocessed the output file to extract the triplets for each sentence. We identify the actors and the links between the nodes through each triplet.

**Algorithm 1** Extraction process from OpenIE 5.0 and OpenIECoreNLP

1: Input: Textual_Requirement, system_name

2: Output: actorsPhase2 and links from all sentences of textual requirement

3: Begin

4: Preprocessing

5: **for** each sentence in the requirement **do**

6:  // Return noun, verb and compound noun lists at index 0, 1 and 2 respectively

7:  *Noun_Verb_CompoundNoun_List* ← Noun_Verb_CompoundNoun(sentence)

8:  **for** each triplet of sentence

9:   // Return *actorsPhase*2 and links at index 0 and 1 respectively

10:   *Actor_Phase2_Links_List* ← Actor_Phase2 (subject, predicate, object)

11:  **end for**

12: **end for**

13: **return** *Actor*_*Phase*2_*Links*_*List*

14: End

### Noun and verb identification

In Algorithm 2, our proposed approach parses each sentence to extract nouns and verbs using the corresponding POS tags. These POS tags are discussed in [51].

**Algorithm 2** Noun_Verb_CompoundNoun

1: Input: Sentence

2: Output: Noun, Verb and Compound Noun Identification

3: Begin

4: *annotated_sentence* ← Annotate(sentence)

5: *tree* ← Parse(*annotated sentence*)

6: **for** each leaf in the *tree* **do**

7: // Identification of noun

8:  **if** (parent.equals(“NN”)||(parent.equals(“NNS”)||(parent.equals(“NNP”)|| (parent.equals(“NNPS”) **then**

9:   *NounList* ← leaf

10: // Identification of verb

11:  **else if** (parent.equals(“VB”)||(parent.equals(“VBD”)||(parent.equals(“VBG”)|| (parent.equals(“VBN”)||(parent.equals(“VBP”)||(parent.equals(“VBZ”) **then**

12:   *VerbList* ← *leaf*

13:  **end if**

14: **end for**

15: Remove stopWordsSymbols from *NounList*

16: *Actorlist* ← Actor_Phase(*NounList*)

17: **for** each leaf in *tree* **do**

18:  **for** each actor in the *Actorlist* **do**

19:   **if** leaf.equalsIgnoreCase(actor)&&leaf.parent(tree).parent(tree).label().equals(“NP”) **then**

20:    Extract tree as compoundtree containing all children of leaf.parent(tree).parent(tree)

21:    **for** each node in the *tree* **do**

22: // Ignore conjunctions, determiners, symbols, hyphens and numbers

23:     **if**!(node.equals(“DT”))&&!(node.equals(“CD”))&&!(node.equals(“HYPH”)) &&!(node.equals(“SYM”))&&!(node.equals(“CC”)) **then**

24:      Set the *compoundNoun* with the values of all leaves

25:     **end if**

26:    **end for**

27:    **if** *cmpnoun*.wordlength > 1 **then**

28:     *compoundNoun* ← *cmpnoun*.toLowerCase()

29:    **end if**

30:   **end if**

31:  **end for**

32: **end for**

33: *Noun*_*Verb*_*CompoundNoun*_*List*.add(*NounList*) // NounList store at index 0

34: *Noun*_*Verb*_*CompoundNoun*_*List*.add(*VerbList*) // VerbList store at index 1

35: // compoundNoun store at index 2

36: *Noun*_*Verb*_*CompoundNoun*_*List*.add(*compoundNoun*)

37: *Noun*_*Verb*_*CompoundNoun*_*List*

38: End

### Actor phase 1

In the use case model, there are the primary and external actors. We have identified both types of actors. The primary and external actors may correspond to some person or system. In our approach, we deal with both kinds of scenarios. Algorithm 3 illustrates the first phase of actor generation. In the first phase, the noun list for each sentence is given as input. We traverse each noun in the list at three levels to identify actors. In the first level, if the word in the noun list lies in the lexical category of noun.person, then it represents an actor. Our approach also semantically identifies the actors. To do this, in the second level, our approach uses the gloss that defines a word. In most cases, this definition also includes example sentences. Our approach checks whether any token in the gloss contains the word “system, instrument, machine, device or equipment”. If the gloss of a word contains any defined token and satisfies one of the following two conditions, then the word represents an actor:

- The word is equal to “system”, “instrument”, “machine”, “device”, “software” or “equipment”
- The original system name does not contain the word

The defined words in the first condition cover the scenario where some compound words represent any system as an actor. However, it does not correspond to the name of the original system. In the second condition, the original system name represents the system for which the use cases and actors are identified.

In the last level, the synset of each word repeats level 1 and level 2. If any synset of a word satisfies level 1 or level 2, then that particular word identifies as an actor.

**Algorithm 3** Generation of Actors Phase 1

1: Input: NounList

2: Output: actorsPhase1

3: Begin

4: *NounList →* lemmatize(*NounList)*

5: **for** each word in the *NounList* **do**

6: // Actor Phase 1 Level 1

7:  **if** word.equals(“noun.person”) **then**

8:   *actorsPhase*1 ← word

9:   *foundperson* ← true

10:  **end if**

11: // Actor Phase 1 Level 2

12:  **if** *foundperson*==false

13:   Get gloss of word

14:   **for** each *token* in the gloss

15:    **if** *foundperson*==lemmatize(*token*)

16:    **if**(*token*.contains(system))||(*token*.contains(instrument))|| (*token*.contains(machine))||(*token*.contains(device))||(*token*.contains(equipment))

17:     **if** word.equals(“system”)||word.equals(“device”)||word.equals(“machine”) ||word.equals(“instrument”)||word.equals(“equipment”)||word.equals(“software”) **then**

18:      *actorsPhase*1 ← word

19:      *foundgloss* ← true

20:     **else if**(!*system*_*name*.*contains*(*word*)) **then**

21:      *actorsPhase*1 ← *word*

22:      *foundgloss* ← *true*

23:     **end if**

24:    **end if**

25:   **end for**

26:  **end if**

27: **end for**

28: // Actor Phase 1 Level 3

29: **if** *foundperson*==false&&*foundgloss*==false **then**

30:  **for** each synset of word **do**

31:   Repeat step 6-26

32:  **for**

33: **end if**

34: **return** *actorsPhase*1

35: End

### Compound noun identification

The compound noun can also represent an actor like bank client. In Algorithm 2, we check the parent node of each noun identified as an actor in Phase 1. If the parent tag is NP then all leaf nodes of the subtree other than those containing conjunctions, determiners, symbols, and numbers represent the compound noun. The conjunctions, determiners, symbols, hyphens and numbers are identified using the POS tags “CC”, “DT”, “SYM”, “HYPH”, and “CD” respectively. Later, these compound nouns are used to identify the actors in phase 2.

### Actor phase 2

In the use case model, the actors should initiate some use cases. To deal with this scenario, we need to incorporate a restriction on actors identified in phase 1 that the actor should be either in the subject for active voice sentences or in the object for passive voice sentences. To do this, Algorithm 4 provides the steps for the identification of actors in Phase 2. It takes the subject, predicate, and object of each triplet as input. We remove the set of symbols from each triplet. These symbols are availale at https://github.com/stanfordnlp/CoreNLP/blob/main/data/edu/stanford/nlp/patterns/surface/stopwords.txt.

Each actor in Phase 1 matched with the subject of the triplet. On a successful match, we have checked firstly, the existence of an actor as a compound or single noun and secondly, ensured it is not equal to the system name. When both the conditions are satisfied, we add the actor from Phase 1 into Phase 2. We have also checked that actor as a single noun is not equal to the words including system, instrument, machine, device, and equipment. These single nouns do not correspond to an actor. We represent the subject noun and object noun using the nouns in the subject and object part of each triplet. More than one noun in the subject or object is separated by the symbol “_”. These subject nouns and object nouns are used as nodes for the formation of a graph.

Our approach also deals with passive voice sentences that contain actors in the object part. If the triplet has an object part with the word “by” followed by an actor in Phase 1, then the sentence is in passive voice. The object part matches with each actor of Phase 1 and all the conditions discussed earlier are applied to identify actors in Phase 2. We convert the object-predicate-subject into subject-predicate-object. To convert this, our approach assigns the noun(s) in the object part to the subject noun and noun(s) in the subject part to the object noun.

Along with actor generation, this algorithm provides the set of links that can be used for the formation of network. The nodes in the network contains subject noun, predicate verb and object noun.

Our approach represents each verb in the predicate as the predicate verb. More than one verb in the predicate is separated by the symbol “_”. We remove the following helping verbs from the predicate verb.

Stop words = {“are”, “be”, “been”, “can”, “could”, “did”, “do”, “does”, “had”, “has”, “have”, “having”, “should”, “was”, “were”, “would”, “will”, “shall”}

It is possible that the predicate verb becomes empty on the removal of the stop words. In this case, our approach finds the first verb in the object which is not a stop word. If we get a verb, then it becomes the predicate verb and the remaining words followed by an extracted verb are the part of the object.

**Algorithm 4** Generation of Actors Phase2

1: Input: subject, predicate and object

2: Output: Actors at phase 2 and links from the subject, predicate and object on sentence level

3: Begin

4: Remove stopwordSymbol from *subject*, *predicate* and *object*

5: prediacteverb ← lemmatize(VerbList_in_predicate).replaceAll(“”, “_”)

6: *predicateverb* ← *predicateverb*.remove(stopwords)

7: **if** *predicateverb*.isEmpty() **then**

8:  *prediacteverb* ← lemmatize(first_nonstopword_verb_in_object)

9:  *object* ← word follows verb in object

10: **end if**

11: **for** each actor in the *ActorList*

12: // Actor identification from active voice sentences

13:  **if** *subject*.contains(actor) **then**

14:   objectnoun ← lemmatize(nounlist_in_object).replaceAll(“”, “_”)

15:   **for** each *compoundNoun* **do**

16:    **if** *compoundNoun*.contains(actor)&&*subject*.contains(*compoundNoun*) **then**

17:     *subjectnoun* ← lemmatize(*compoundNoun*).replaceAll(“”,“_”)

18:     **if**!*subjectnoun*.equals(*system*_*name*) **then**

19:      *actorsPhase*2 ← *subjectnoun*

20:      *foundsubjectactor* ← true

21:      else

22:      *subjectnoun* & *objectnoun* ← “”

23:     **end if**

24:    **end if**

25:   **for**

26:   **if**! *compoundNoun* **then**

27:    *subjectnoun*← lemmatize(NounList_in_subject).replaceAll(“”, “_”)

28:    **if** *subjectnoun*!=system, instrument, machine, device, equipment &&!*subjectnoun*.contains(*system*_*name*) **then**

29:     Repeat step 19 to 22

30:    **end if**

31:   **end if**

32:  **end if**

33: // Actor identification from passive voice sentences

34:  **if** *object*.contains(“by”)&&wordsfollowsBy.contains(actor) **then**

35:   **if** *objectby* ← wordsfollowsBy in object

36:   *objectnoun* ← lemmatize(NounList_in_subject).replaceAll(“”, “_”)

37:   **for** each *compoundNoun*

38:    **if** *compoundNoun*.contains(actor)&&*objectby*.contains(*compoundNoun*) **then**

39:     *subjectnoun* ← lemmatize(*compoundNoun*).replaceAll(“”,“_”)

40:     **if**!*subjectnoun*.equals(*system*_*name*) **then**

41:      *actorsPhase*2 ← *subjectnoun*

42:      *foundobjectactor* ← true

43:      else

44:      *subjectnoun* & *objectnoun* ← “”

45:     **end if**

46:    **end if**

47:   **end for**

48:   **if**!*compoundNoun* **then**

49:    *subjectnoun* ← lemmatize(NounList_in_objectby).replaceAll(“”,“_”)

50:    **if** *subjectnoun*!=system, instrument, machine, device, equipment &&!*subjectnoun*.contains(*system*_*name*) **then**

51:     Repeat step 41 to 44

52:    **end if**

53:   **end if**

54:  **end if**

55: **end for**

56: **if** *foundsubjectactor*==false && *foundobjectactor*==false **then**

57:  Repeat steps 14 & 27

58: **end if**

59: // Set of links for the formation of network

60: **if** *subjectnoun*!=“” && *objectnoun*!=“” && *predicateverb*!=“” **then**

61:  *link*1 ← *subjectnoun* connects to *predicateverb*

62:  *link*2 ← *predicateverb* connects to *objectnoun*

63:  *links* ← list of all *link*1 and *link*2

64: **end if**

65: *Actor*_*Phase*2_*Links*_*List*_*Sentence*.add(*actorsPhase*2) // Actors store at index 0

666: *Actor*_*Phase*2_*Links*_*List*_*Sentence*.add(*links*) // Links store at index 1

67: *Actor*_*Phase*2_*Links*_*List*_*Sentence*

68: End

### Network formation

**Fig 4:**
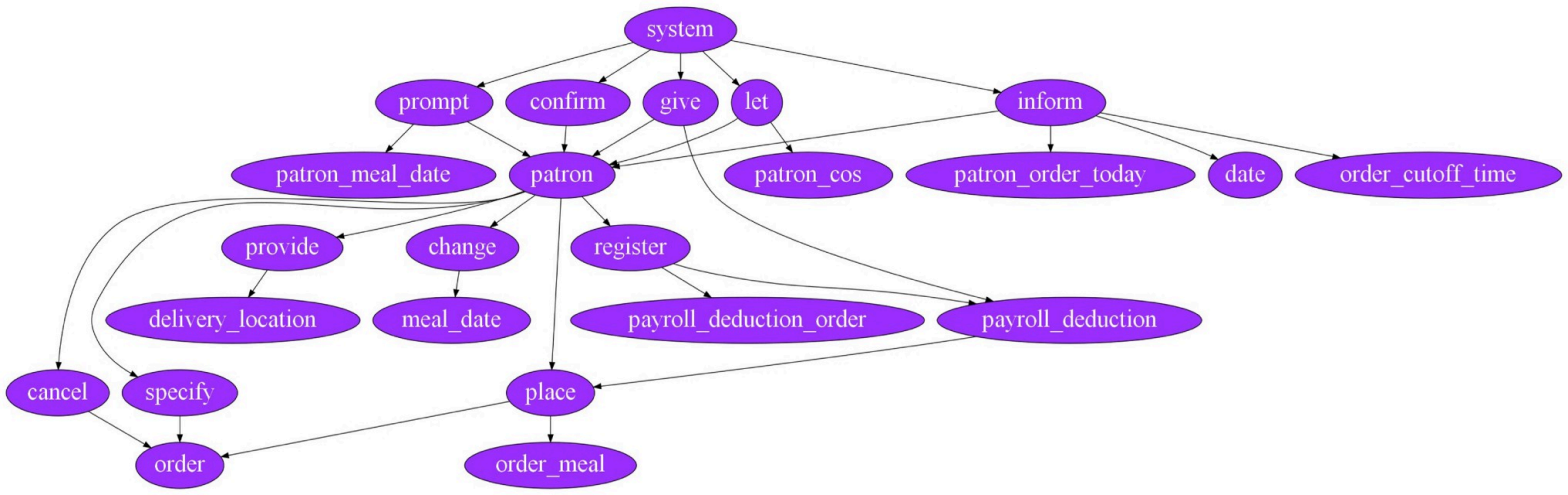
Network extraction from textual requirement.

Our approach produced the network using the subject noun, predicate verb, and object noun for all triplets in the textual requirement. These nodes are connected through a directed link. The network does not contain any duplicate nodes. In the network, the subject noun connects with the predicate verb, and the predicate verb connects with the object noun for each triplet. Fig 4, shows an example of a network generated from the textual requirement.

### Actor phase 3

In the final phase of actor identification, we have checked that the actor should connected with some other nodes in the graph. Therefore, all the actors identified in Phase 2 with degree greater than zero represent the final actor.

### Use cases and relationships

A use case should be the combination of nouns and verbs. The nodes at depth 1 and depth 2 from the actor contain verbs and nouns respectively. Therefore, we traverse the ego network of each final actor till depth 2. All the nodes at depth 1 with the outgoing edges towards the nodes at depth 2 represent use cases as listed in Algorithm 5. Each connecting node till depth two from an actor in the graph represents the relationship.

**Algorithm 5** Generation of Actors Phase 3, Use Case Identification & Network Formation

1: Input: actorsPhase2 and set of links

2: Output: actorsPhase3, Use Cases and Network

3: Begin

4: // Network Formation

5: **for** each link1 and link2

6:  Make a network

7:**for**

8: // Generation of Actors Phase 3

9: **for** all nodes in the graph

10:  **for** each actor in *actorsPhase*2

11:   **if**(outdegree>0)&&node.contains(actor) **then**

12:    *actorsPhase*3 ← node

13:   **end if**

14:  **for**

15: **for**

16: // Use Case Identification

17: **for** each actor in *actorsPhase*3

18:  Traverse the ego network of actor till depth 2

19:  All the nodes at depth 1 with the outgoing edges towards the nodes at depth2 represents use cases.

20: // Relationships

21:  *relationships* ← actor connected to use cases

22: **end for**

23: Display *actorsPhase*3, use cases, *relationships*

24: End

## Experimental setup

**Fig 5:**
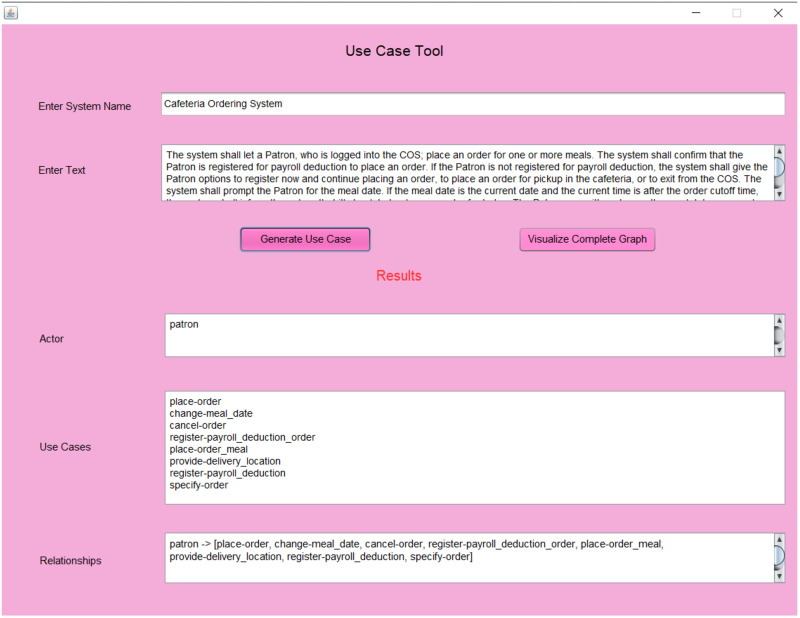
Graphical user interface of proposed approach.

The algorithms are implemented in Java language using Netbeans IDE. These experiments are performed on Dell PC with 8 GB RAM, i7-8550 CPU, with Windows 10 operating system. The NLP techniques are implemented using Stanford CoreNLP 4.3.2 (https://stanfordnlp.github.io/CoreNLP/). We have used Open IE 5.0 (https://github.com/dair-iitd/OpenIE-standalone) and OpenIE CoreNLP (https://stanfordnlp.github.io/CoreNLP/openie.html) for triplet extraction. All the tasks with WordNet 3.0 (https://wordnet.princeton.edu/) are implemented using JWI 2.4.0 (https://projects.csail.mit.edu/jwi/). We have used JGraphT 1.3.0 (https://jgrapht.org/) and Graphviz 2.50.0 (https://graphviz.org/) for the techniques of the Network Science. Fig 5, shows the graphical user interface of our proposed approach.

We applied our proposed approach to five literature-based case studies [11]. It includes an ATM System, Cafeteria Ordering System, Library System, Assembly System, and Time Monitor Software System. We have evaluated the results using Precision, Recall and F-Measure.

## Results analysis and discussion

We have evaluated the results in two categories for all case studies. In the first category, the results are evaluated using the manual actors and use cases identified in Al-Hroob et al. [11]. In the second category, we have refined the manual results identified in Al-Hroob et al. [11]. It has been observed that most of the manually identified use cases from the case studies contain only a single word i.e verb [11]. However, a use case should contain both the noun and verb. We have also manually identified some additional actors and use cases for an Assembly System Case Study.

As in the second category the manual use case contains both the noun and verb, therefore, only those use cases are true-positives that contain both the noun and verb and are similar to the refined manual results. We revaluate the Al-Hroob et al. [11] approach for Category 2.

We compare the results produced by our proposed approach with Al-Hroob et al. [11] approach for both the categories.

Table 1, shows the results of our proposed approach and Al-Hroob et al. [11] for the ATM System Case Study. The first two rows in Table 1 show the actors and all the subsequent rows show the use cases. The third column contains actors and use cases that are refined manually.

**Table 1:** Actors & use cases for case study 1.

Element	Manual	Refined Manual	Proposed Approach	Al-Hroob et al. [11] Approach
**Actor(s)**	Bank_Client	Bank_Client	bank_client	Application
			client	Client
**Use Cases**	Deposit	Deposit amount	gain-access_account	Cover
	Withdraw	Withdraw amount	withdraw-amount_account	Deposit
	Review	Review transaction	deposit-amount	Gain
	Access	Access account	deposit-account	Withdraw
	Inform	Inform user	gain-access_account_identification	Have
			withdraw-amount	Include
			review-transaction	Inform
			gain-access_identification	Review
			withdraw-save_account	
			gain-access	
			withdraw-balance	
			withdraw-fund	
			gain-identification_number	

Our proposed approach correctly identifies 4 out of 5 manually identified use cases and one actor for both categories. Our proposed approach has 14 true positives for the first category that include “bank_client, client, gain-access_account, withdraw-amount_account, deposit-amount, deposit-account, gain-access_account_identification, withdraw-amount, review-transaction, gain-access_identification, withdraw-save_account, gain-access, withdraw-balance” and “withdraw-fund”. The results indicate the use case “gain-identification_number” as a false positive and “inform” as a false negative.

In the second category, we analyze the results using the refined manual. Our approach has 8 true positives that include “bank_client, client, gain-access_account, withdraw-amount_account, deposit-amount, withdraw-amount, review-transaction” and “withdraw-fund”. All the remaining results are false positives. Our approach does not identify the “inform user” use case. However, Al-Hroob et al. [11] has only 1 true positive i.e “client”, 5 false negatives, and 9 false positives in the second category.

Some of the use cases are almost identical to each other with minor differences. However, they resemble the manually identified use cases. Therefore, we count all of them as true positives.

Table 2 shows the results of our proposed approach and the Al-Hroob et al. [11] approach for the Cafeteria Ordering System Case Study.

**Table 2:** Actors & use cases for case study 2.

Element	Manual	Refined Manual	Proposed Approach	Al-Hroob et al. [11] Approach
**Actor(s)**	Patron	Patron	patron	Patron
				System
**Use Cases**	Log	Log_COS	place-order	Cancel
	Place_an_order	Place_an_order	change-meal_date	Change
	Register	Register_payroll_deduction	cancel-order	Inform
	Place_order_for_pickup	Place_order_for_ pickup	register-payroll_deduction_order	Let
	Exit from COS	Exit from COS	place-order_meal	Place
	Change_meal_date	Change_meal_date	provide-delivery_location	Provide
	Cancel_order	Cancel_order	register-payroll_deduction	
	Specify_Option	Specify_Option	specify-order	
	Provide_ valid_delivery_location	Provide_ valid_delivery_location		

Table 2, indicates all results produced by our proposed approach as true positive for both categories. Our proposed approach has two false negatives for the first category including “Log” and “Exit from COS” use cases.

In the second category, our proposed approach has another false negative that is “Place_order_for_pickup”. However, Al-Hroob et al. [11] has only 1 true positive i.e “patron”, 9 false negatives, and 7 false positives in the second category.

Table 3, shows the results of our proposed approach and the Al-Hroob et al. [11] approach for the Library System Case Study.

**Table 3:** Actors & use cases for case study 3.

Element	Manual	Refined Manual	Proposed Approach	Al-Hroob et al. [11] Approach
**Actor(s)**	Customer	Customer	customer	Book
				Customer
				Item
				Library
**Use Cases**	Issue_membership_ card	Issue_membership_card	know-member	Extend
	Borrow	Borrow item	scan-bar_code_reader	Has
	Reserve	Reserve item	issue-membership_card_ member_ number	Borrow
	Renew	Renew item	issue-membership_card	Reserve
	Extend	Extend loan	borrow-item	Search
	Scan	Scan membership number		Shows
	Enter	Enter membership number		
	Search	Search item		

Our proposed approach attains one actor and 4 use cases as true positive for both categories including “scan-bar_code_reader, issue-membership_card_member_number, issue-membership_card” and “borrow-item”. The remaining use case is false positive, and five use cases as false negatives. However, Al-Hroob et al. [11] has only 1 true positive i.e “customer”, 9 false positives, and 8 false negatives in the second category.

Table 4, shows the results of our proposed approach and the Al-Hroob et al. [11] approach for the Assembly System Case Study. The first five rows in Table 4 show the actors and all the subsequent rows show the use cases.

**Table 4:** Actors & use cases for case study 4.

Element	Manual	Refined Manual	Proposed Approach	Al-Hroob et al. [11]
**Actor(s)**	User	User	robot	User
		Vision System	vision_system	Belt
		Robot	arm_robot	Robot
			user	Part
				System
**Use Cases**	Put	Put kind_Part	pick-belt	Conveys
		Enter Sensor Zone	inform-robot	Enter
		Inform belt	put-kind_part	Senses
		Recognize Type	pick-part	Informs
		Inform Robot	put-kind	Pick
		Pick part	put-kind_cup	Puts
		Place cup	recognize-type_part	Recognizes
		Place dish	place-arm	
			place-cup	
			put-kind_dish	
			recognize-type	
			place-dish	
			inform-belt	

Our proposed approach correctly identifies all use cases and actors for Category 1. Our approach has 5 true positives including “user, put-kind_part, put-kind, put-kind_cup” and “put-kind_dish”. All the remaining are false positives.

In the second category, some additional actors and use cases are manually identified. Our approach has 11 true positives including “robot, vision_system, user, inform-robot, put-kind_part, pick-part, recognize-type_part, place-cup, recognize-type, place-dish” and “inform-belt”. Our approach has one false negative for Category 2 i.e “Enter Sensor Zone”. However, Al-Hroob et al. [11] has 2 true positives i.e “user” and “robot”, 10 false positives, and 9 false negatives in the second category.

Table 5, shows the results of our proposed approach and the Al-Hroob et al. [11] approach for the Time Monitor Software System Case Study. The first four rows in Table 5 show the actors and all the subsequent rows show the use cases.

**Table 5:** Actors & use cases for case study 5.

Element	Manual/Refined Manual	Proposed Approach	Al-Hroob et al. [11]
**Actor(s)**	Developer	www_browser	Developers
	Manager	manager	Manager
		developer	Record
			Task
**Use Cases**	Use_Browser	define-week	Use
	Analyse_Time_Record	define-task	Allow
	Define_Task	define-work	Analyse
		define-week_monday	Define
		analyse-timestamp_record	Involve
		store-timestamp_record	
		store-database	
		work-task	
		use-www_browser	

The manual and refined results are the same for case study 5. However, the evaluation criteria for the use case are different. As in the first category we are using the criteria of Al-Hroob et al. [11], only the verb is compared to identify a use case. Moreover, in the second category, the use case must contain both the noun and verb and should also be similar to the refined manually identified use cases. Therefore, our proposed approach has 8 true positives for Category 1 that include “manager, developer, define-week, define-task, define-work, define-week_Monday, analyse-timestamp_record” and “use-www_browser”. Also, our approach has 6 true positives for category 2 that include “manager, developer, define-task, define-work, analyse-timestamp_record” and “use-www_browser”. Our approach identifies all actors and use cases successfully for both categories. However, Al-Hroob et al. [11] has 2 true positives i.e “Developers” and “Manager”, 7 false positives, and 3 false negatives in the second category.

Tables 6 and 7 summarizes the true positive, false positive and false negative results of our proposed approach and Al-Hroob et al. [11] for both categories.

**Table 6:** True positive, false positive, false negative for category 1.

Case Studies	Proposed Approach			Al-Hroob et al. [11]		
	True Positive	False Positive	False Negative	True Positive	False Positive	False Negative
Case Study 1	14	1	1	5	5	1
Case Study 2	9	0	2	5	3	4
Case Study 3	5	1	5	5	5	4
Case Study 4	5	12	0	2	10	0
Case Study 5	8	4	0	5	4	0

**Table 7:** True positive, false positive, false negative for category 2.

Case Studies	Proposed Approach			Al-Hroob et al. [11]		
	True Positive	False Positive	False Negative	True Positive	False Positive	False Negative
Case Study 1	8	7	1	1	9	5
Case Study 2	9	0	3	1	7	9
Case Study 3	5	1	5	1	9	8
Case Study 4	11	6	1	2	10	9
Case Study 5	6	6	0	2	7	3

Table 8, shows the Precision, Recall, and F-Measure for all case studies. These results are analyzed using the manual actors and use cases for Category 1. The result demonstrates that on average, our approach outperforms the Al-Hroob et al. [11] approach. Our approach has better Precision and F-Measure for all case studies than the Al-Hroob et al. [11] approach. Both approaches have a similar recall for case studies 4 and 5. Our proposed approach improves the recall for case studies 1 and 2. However, only for case study 3 does the Al-Hroob et al. [11] approach have more recall.

**Table 8:** Precision, recall, f-measure for category 1.

Case Studies	Proposed Approach			Al-Hroob et al. [11]		
	Precision	Recall	F-Measure	Precision	Recall	F-Measure
Case Study 1	93.33%	93.33%	93.33%	50%	83%	63%
Case Study 2	100%	81.8%	89.98%	63%	56%	59%
Case Study 3	83.33%	50%	62.49%	50%	56%	53%
Case Study 4	29.4%	100%	45.44%	17%	100%	29%
Case Study 5	66.67%	100%	80%	56%	100%	71%
Average	74.5%	85%	74.2%	47.2%	79%	55%

Table 9, shows the Precision, Recall, and F-Measure for all case studies. These results are analyzed using the refined manual actors and use cases for Category 2. Our proposed approach significantly outperforms the Al-Hroob et al. [11] approach for all case studies. On average, our approach has 70.27% precision, 81% recall, and 71.478% F-measure.

**Table 9:** Precision, recall, f-measure for category 2.

Case Studies	Proposed Approach			Al-Hroob et al. [11]		
	Precision	Recall	F-Measure	Precision	Recall	F-Measure
Case Study 1	53.33%	88.89%	66.66%	10%	16.67%	12.5%
Case Study 2	100%	75%	85.7%	12.5%	10%	11.11%
Case Study 3	83.33%	50%	62.49%	10%	11.11%	10.52%
Case Study 4	64.705%	91.7%	75.87%	16.67%	18.18%	17.39%
Case Study 5	50%	100%	66.67%	22.22%	40%	28.57%
Average	70.27%	81%	71.478%	14.278%	19.192%	16.018%

Table 10, shows the False Discovery Rate and Miss Rate for all case studies. These results are analyzed using the manual actors and use cases for Category 1. It is observed that our proposed approach significantly outperforms the Al-Hroob et al. [11] approach for all case studies. On average, our approach has 25.45% False Discovery Rate, and 14.97% Miss Rate.

**Table 10:** False discovery rate and miss rate for category 1.

Case Studies	Proposed Approach		Al-Hroob et al. [11]	
	False Discovery Rate	Miss Rate	False Discovery Rate	Miss Rate
Case Study 1	6.67%	6.67%	50%	17%
Case Study 2	0%	18.20%	37%	44%
Case Study 3	16.67%	50%	50%	44%
Case Study 4	70.6%	0%	83%	0%
Case Study 5	33.33%	0%	44%	0%
Average	25.45%	14.97%	53%	21%

Table 11, shows the False Discovery Rate and Miss Rate for all case studies. These results are analyzed using the refined manual actors and use cases for Category 2. It is observed that our proposed approach significantly outperforms the Al-Hroob et al. [11] approach for all case studies. On average, our approach has 29.73% False Discovery Rate, and 19% Miss Rate.

**Table 11:** False discovery rate and miss rate for category 2.

Case Studies	Proposed Approach		Al-Hroob et al. [11]	
	False Discovery Rate	Miss Rate	False Discovery Rate	Miss Rate
Case Study 1	46.67%	11.11%	90%	83.33%
Case Study 2	0%	25%	88%	90%
Case Study 3	16.67%	50%	90%	88.89%
Case Study 4	35.30%	8.30%	83%	81.82%
Case Study 5	50%	0%	78%	60%
Average	29.73%	19%	85.72%	80.81%

We have observed from the results that the missing use cases are due to the missing triplets, or else the triplet does not contain an actor in the subject. Whether the sentence is either written in active or passive voice, our approach identifies the actor from the subject in phase 2. We have converted object-predicate-subject to subject-predicate-object for passive voice sentences.

## Threats to validity

In this section, we discuss the threats to the validity of our proposed approach.

### Internal validity

To provide correct results, the sentences need to be in active or passive voice with the actor in the subject or object part.

### Construct validity

It is required to include all libraries as discussed in the Experimental Setup section for the proper working of the approach.

## Conclusion

This paper provides a systematic literature review for the generation of use cases from natural language requirements. We have observed from the literature that most of the approaches are either semi-automated or require restricted natural language/ formalism.

Therefore, in this paper, an automated approach is proposed for the generation of the actor, use cases, and their relationships without depending on formalism/ restricted natural language. Our approach uses NLP techniques and network science. It generates both primary and external actors. Our approach generates the actors in three phases. In the first two phases, the natural language processing techniques are involved and in the third phase, it is generated using network science. The use cases are identified from the network using Network Science. The node values are set using NLP techniques. Our approach comprises a set of five algorithms. The first algorithm discusses the extraction of actors and the links through each triplet of each sentence. The second algorithm identifies nouns, verbs, and compound nouns. The third algorithm generates the actor at Phase 1. These actors are refined in Phase 2 using the fourth algorithm. The last algorithm generates the final actor, use cases, and relationships.

Our approach is validated on five case studies. Our proposed approach outperforms in comparison with the existing approach. The results are analyzed in two categories for all case studies. On average, our approach achieves 74.5% precision, 85% recall, 74% F-Measure, 25.45% False Discovery Rate, and 14.97% Miss Rate for the first category. Moreover, on average, our approach has 70% precision, 81% recall, 71.5% F-measure, 29.73% False Discovery Rate, and 19% Miss Rate for the second category.

In the future, we will extend the approach to link the actors with those sentences that contain use cases without the presence of actors.
